# Dopamine Transporters in Striatum Correlate with Deactivation in the Default Mode Network during Visuospatial Attention

**DOI:** 10.1371/journal.pone.0006102

**Published:** 2009-06-30

**Authors:** Dardo Tomasi, Nora D. Volkow, Ruiliang Wang, Frank Telang, Gene-Jack Wang, Linda Chang, Thomas Ernst, Joanna S. Fowler

**Affiliations:** 1 National Institute on Alcohol Abuse and Alcoholism, Bethesda, Maryland, United States of America; 2 National Institute on Drug Abuse, Bethesda, Maryland, United States of America; 3 Medical Department, Brookhaven National Laboratory, Upton, New York, United States of America; 4 Department of Medicine, University of Hawaii, Honolulu, Hawaii, United States of America; University of Minnesota, United States of America

## Abstract

**Background:**

Dopamine and dopamine transporters (DAT, which regulate extracellular dopamine in the brain) are implicated in the modulation of attention but their specific roles are not well understood. Here we hypothesized that dopamine modulates attention by facilitation of brain deactivation in the default mode network (DMN). Thus, higher striatal DAT levels, which would result in an enhanced clearance of dopamine and hence weaker dopamine signals, would be associated to lower deactivation in the DMN during an attention task.

**Methodology/Principal Findings:**

For this purpose we assessed the relationship between DAT in striatum (measured with positron emission tomography and [^11^C]cocaine used as DAT radiotracer) and brain activation and deactivation during a parametric visual attention task (measured with blood oxygenation level dependent functional magnetic resonance imaging) in healthy controls. We show that DAT availability in caudate and putamen had a negative correlation with deactivation in ventral parietal regions of the DMN (precuneus, BA 7) and a positive correlation with deactivation in a small region in the ventral anterior cingulate gyrus (BA 24/32). With increasing attentional load, DAT in caudate showed a negative correlation with load-related deactivation increases in precuneus.

**Conclusions/Significance:**

These findings provide evidence that dopamine transporters modulate neural activity in the DMN and anterior cingulate gyrus during visuospatial attention. Our findings suggest that dopamine modulates attention in part by regulating neuronal activity in posterior parietal cortex including precuneus (region involved in alertness) and cingulate gyrus (region deactivated in proportion to emotional interference). These findings suggest that the beneficial effects of stimulant medications (increase dopamine by blocking DAT) in inattention reflect in part their ability to facilitate the deactivation of the DMN.

## Introduction

Dopamine (DA) neurotransmission is believed to play a central role in attention [1]. It is recognized that disruption of brain DA activity contributes to inattention and that stimulant medications (methylphenidate, amphetamine), which enhance DA activity by targeting the dopamine transporters (DAT) are beneficial in the treatment of inattention [2]. However, the mechanisms through which DA modulates attentional processes are not well understood.

Positron emission tomography (PET) technology can be used to directly measure markers of DA activity. Likewise, functional magnetic resonance imaging (fMRI) allows mapping of brain activation in brain regions modulated by DA [3]. Consequently, PET-fMRI studies make it possible to evaluate the hypothesis that DA neurotransmission can change regional blood oxygenation level dependent (BOLD) signals by modulating neuronal activity [4]. Such a strategy was recently used to evaluate the relationship between DA synthesis in ventral striatum and BOLD signals in prefrontal cortex in response to a positive reward [5], [6] and a working memory task [7].

Multiple brain regions have been implicated in attention [8], [9]. Two attention networks have been proposed on the basis of findings from brain imaging studies; a dorsal one that is involved in purposeful attention (top-down) and a ventral one that reorients attention in response to salient stimuli [10] that might lead to distractions when performing a task [11]. The ventral pathway appears to be preferentially active when individuals are not focused on the external environment and to be deactivated when engaged in purposeful cognitive tasks [12], [13]. In patients with attention deficit hyperactivity disorder (ADHD), stimulant medications (i.e amphetamine and methylphenidate) that block DAT and enhance DA signaling are widely used therapeutically to improve attention [2], [14]. We had shown both in subjects with ADHD and in healthy controls a positive correlation between DAT availability in striatum and inattention [15]. Inasmuch as DAT is the main mechanism for regulating extracellular DA [16], we postulated that the positive association of DAT with inattention reflected lower and shorter DA signals in subjects with higher DAT. Because dopamine is a neurotransmitter that is postulated to enhance task specific signaling while decreasing background neuronal activity [17] we hypothesized that DAT levels would modulate deactivation of the default mode network (DMN). Specifically we hypothesized that subjects with high DAT availability would show attenuated DMN deactivation during an attention task.

To test this hypothesis we used PET and [^11^C]cocaine to measure DAT, and BOLD-fMRI to measure brain activation during a parametric visual attention (VA) task [18] in healthy controls. We specifically hypothesized that striatal DAT availability would correlate negatively with BOLD signals in regions deactivated by the attention-demanding task (VA). This hypothesis was based on the assumption that high DAT levels would result in lower concentration of extracellular DA [19] reducing the ability to deactivate the DMN during the VA task. Thus higher DAT would result in activation rather than deactivation of DMN regions and greater activation of regions from the dorsal network to compensate for the interference of increased task-irrelevant neural processing in DMN regions [17]. We also hypothesized an interaction with cognitive load increases such that higher DAT availability in striatum would be associated with blunted deactivation of DMN regions.

## Materials and Methods

### Subjects

Fourteen healthy, non-smoking, right-handed men (age 36±5 years, education: 16±2 years) participated in the study. All participants provided written informed consent as approved by the Institutional Review Board at Brookhaven National Laboratory. Subjects were screened carefully with a detailed medical history, physical and neurological examinations, EKG, breath carbon monoxide, blood tests, and urine toxicology for psychotropic drug to ensure the subjects were healthy at the time of the study. Subjects were included in the study if they were a) 18 to 50 years old, and b) able to understand and give informed consent. Subjects were excluded if they had c) urine positive for psychotropic drugs (cocaine, phencyclidine, benzodiazepines, cannabis, opiates, barbiturates and inhalants); d) present or past history of dependence on alcohol or other drugs of abuse (except for nicotine and caffeine); or e) present or past history of neurological or psychiatric disorder; f) used psychoactive (i.e. opiate analgesics, stimulants, or sedatives) or g) prescription (non-psychiatric; i.e. antihistamines) medications in the past month, or h) medical conditions that may alter cerebral function; i) cardiovascular and metabolic diseases; j) history of head trauma with loss of consciousness of more than 30 minutes; k) history of sleep disorders; or l) contraindications for MRI (metallic or electronic implants, metallic tattoos in the neck/head, claustrophobia).

### PET imaging

Subjects underwent PET scans with [^11^C]cocaine radiotracer to measure DAT availability in the brain. PET images were acquired in 3D mode using a Siemens HR+ tomograph with 4.5 mm isotropic spatial resolution. Each sequential dynamic scan started immediately after intravenous injection of 4 to 10 mCi of [^11^C]cocaine (specific activity >0.4 Ci/µmol at time of injection) for a total of 60 minutes of scanning [20].

### Analysis of PET Images

**Figure 1:**
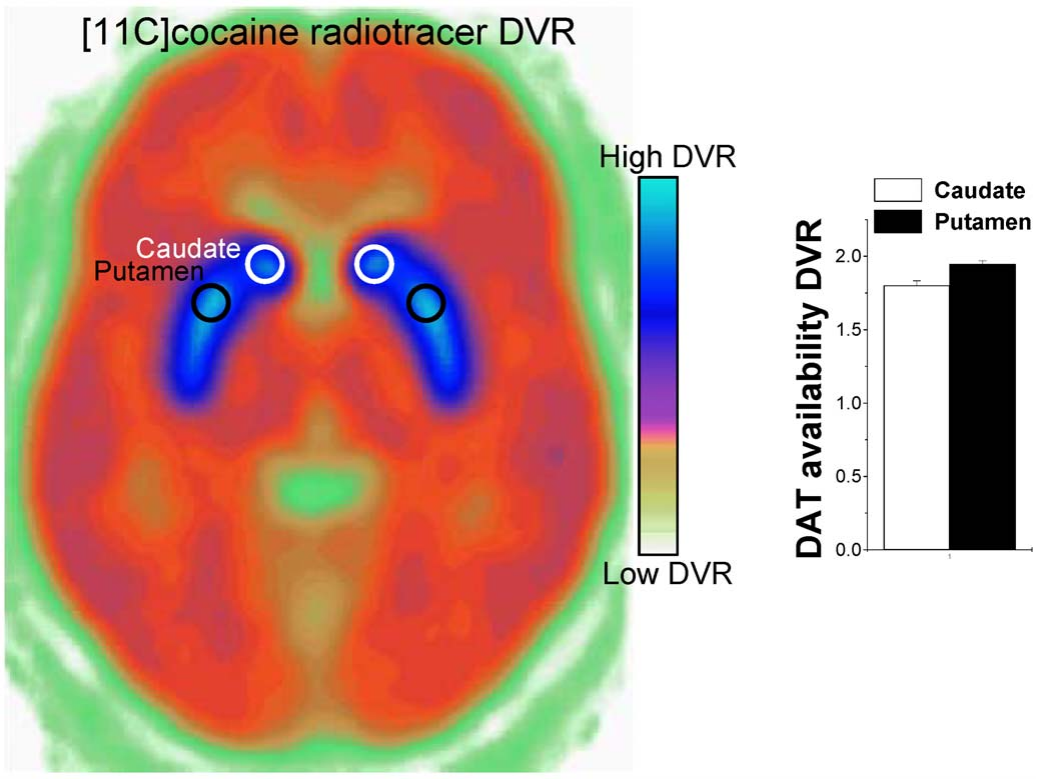
Dopamine transporter (DAT) distribution. Averaged DVR [^11^C]cocaine image at the level of the striatum and ROI measures of DAT availability in caudate and putamen. Values represent means±standard deviations across 14 healthy men.

Regions of interest (ROI) in the striatum (caudate and putamen) and in the cerebellum were defined directly from the [^11^C]cocaine images as previously described [20]. Briefly the ROIs were identified and selected on summed images, resliced along the intercommisural plane (AC-PC line). The bilateral caudate and putamen were measured on 4 planes and the cerebellum in 2 planes. These regions were then projected to the dynamic scans to obtain concentrations of C-11 vs. time, which were used to calculate the distribution volumes (DV) using a graphical analysis technique for reversible systems that does not require arterial blood sampling [21]. We computed the ratio of the DV (DVR) in caudate and putamen to that in the cerebellum to estimate DAT availability (Fig. 1).

### Functional MRI: The VA paradigm

**Figure 2:**
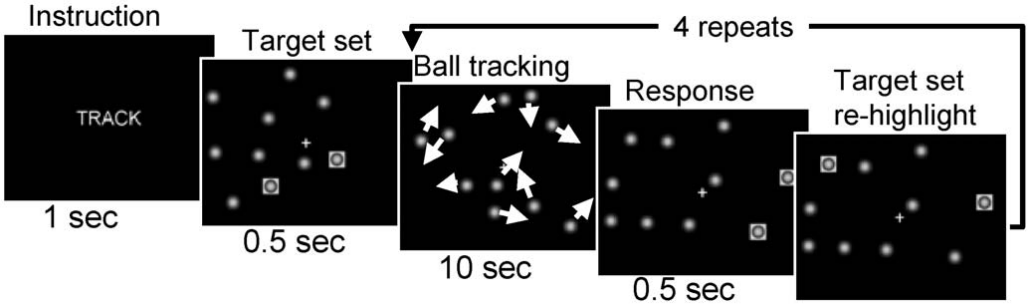
Visual attention (VA) paradigm. During “TRACK” epochs of the 2-ball tracking task, subjects tracked the target ball set that was briefly highlighted after the instruction. Then all 10 balls moved with a random motion for 10 seconds and the subjects *tracked* the target balls until the balls stop moving. Then subjects *responded* by pressing a button if the highlighted balls were those they were tracking. Finally the target set was re-highlighted to refocus the subjects' attention on the balls. This *track* and *respond* sequence was repeated 5 times during the 1-minute long “TRACK” epochs.

We used a blocked VA paradigm that requires sustained attention and visual indexing [22], a pure attention process that allows tagging a small number of visual objects in the visual field for fast access of subsequent attention processing, and has been described previously [18], [23]–[26]. Briefly, the attention or “TRACK” epochs have a duration of 1 minute and are composed of five consecutive “tracking” and respond periods in which, two, three, or four out of ten balls are briefly highlighted (target balls); then all balls start to move and the subjects' task was to fixate on the center cross and track the target balls as they move randomly across the central visual field. At the end of “tracking” periods, the balls stop moving and a new set of balls is highlighted and the subjects' were instructed to press a button if the highlighted balls were the target balls (Fig. 2). The control or “DO NOT TRACK” epochs were composed of five consecutive 1 minute long “resting” periods, in which no balls were highlighted and the subjects were instructed to view the 10 balls passively while they were moving and stopping in the same manner as during “TRACK” epochs. Thus, each task (2-, 3-, and 4-balls) was composed by three consecutive “TRACK” - “DO NOT TRACK” epoch cycles and took 6 minutes and 10 seconds. The tasks were presented to the subjects on MRI-compatible goggles connected to a personal computer. All response button events during stimulation were recorded to determine reaction time (RT) and performance accuracy. A trigger signal from the scanner was used to synchronize the paradigm with fMRI acquisition. This VA task activates parietal and occipital cortices, and DA-regulated regions (thalamus, prefrontal cortex, and the cerebellum), commonly involved in visual search [27], attention to visual motion [28], sustained attention [29], [30], selective attention [31], [32], object recognition [33], and orienting VA [34].

### Functional MRI: data acquisition

Subjects underwent MRI in a 4-Tesla whole-body Varian/Siemens MRI scanner. A T2*-weighted single-shot gradient-echo planar imaging (EPI) sequence with ramp-sampling (TE/TR = 20/1600 ms, 4-mm slice thickness, 1-mm gap, 35 coronal slices, 64×64 matrix size, 3.1×3.1 mm in-plane resolution, 90°-flip angle, 231 time points, 200.00 kHz bandwidth) covering the whole brain was used to collect functional images with BOLD contrast. Padding was used to minimize motion. Subject motion was monitored immediately after each fMRI run using a k-space motion detection algorithm [35] written in IDL (ITT Visual Information Solutions, Boulder, CO). Earplugs (28 dB attenuation of sound pressure level; Aearo Ear TaperFit 2; Aearo Co.) and headphones (30 dB attenuation of sound pressure level; Commander XG MRI Audio System, Resonance Technology inc.) and a “quiet” acquisition approach were used to minimize the interference effect of scanner noise during fMRI [36]. Anatomical images were collected using a T1-weighted 3D-MDEFT sequence [37] (TE/TR = 5/15 ms, 0.94×0.94×1.00 mm^3^ spatial resolution, axial orientation, 256 readout and 192×96 phase-encoding steps, 16 minutes scan time) and a modified T2-weigthed Hyperecho sequence [38] (TE/TR = 0.042/10 seconds, echo train length = 16, 256×256 matrix size, typically 30 coronal slices, 0.86×0.86 mm^2^ in-plane resolution, 5 mm thickness, no gap, 2 min. scan time), and reviewed to rule out gross brain morphological abnormalities.

### fMRI analysis

EPI reconstruction was performed using an iterative phase correction method that produced minimal signal-loss artifacts [39]. The first four imaging time points were discarded to avoid non-equilibrium effects in the fMRI signal. The statistical parametric mapping package SPM2 (Welcome Department of Cognitive Neurology, London UK) was used for realignment (head motion was less than 1-mm translations and 1°-rotations for all scans), spatial normalization (using a 12-parameters affine transformation with medium regularization and 16-nonlinear iterations and voxel size of 3×3×3 mm^3^), and smoothing (using an 8-mm FWHM Gaussian kernel). Note that spatial normalization to the Talairach frame of reference was carried out using a modified version of the standard SPM2 EPI template, which was modulated by the average EPI signal intensity across subjects to minimize the effect of brain regions exhibiting strong susceptibility-related signal-loss artifacts during EPI at 4 Tesla. This customized EPI template minimizes spurious geometric distortions during spatial normalization of EPI datasets collected at 4-Tesla using TE = 20 ms. The general linear model in SPM2 and a blocked design with band-pass filtering (the canonic hemodynamic response was used for low-pass filtering; a frequency cut-off = 1/256 Hz was used for high-pass filtering) were used to calculate the activation maps for each condition (2-, 3-, and 4-ball tracking) and subject.

### Statistical analyses: PET-fMRI

We evaluated the effect of DAT on BOLD-fMRI signals through multiple regression analyses. Specifically, the estimated BOLD signal maps for each trial and subject were included in a multiple regression random-effects model in SPM2 with three regressors: 1:(Main VA) a constant regressor modeling the average BOLD amplitude produced by the VA task; 2:(VA-load) a linear regressor (−1, 0, 1), modeling the differential BOLD amplitude produced by the three different cognitive load levels (2-, 3-, and 4-ball tracking); and 3:(DAT) a regressor reflecting the DAT availability measures in the striatum (caudate or putamen). Independent analyses were conducted for DAT measures in caudate and putamen. The continuous random field calculation implemented in SPM2 was used to correct the significance of the activation clusters for multiple comparisons. Brain activation and deactivation clusters with at least 20 voxels (540 mm^3^) and *p*<0.05 (corrected for multiple comparisons) were considered significant.

### Functional ROI-analyses

Brain activation and deactivation clusters were further evaluated with region-of-interest (ROI) analyses to identify outliers that might influence strongly correlation analyses, and to obtain average signal values in a volume comparable to the image smoothness (e.g. resolution elements, or “resels” [40]) rather than single-voxel peak values. The volume of the imaging resels obtained during the second-level SPM analysis was near cubic with a Cartesian full-width-half-maximum (FWHM) = 12.5 mm, 13.1 mm, 13.1 mm. Thus, 9-mm isotropic masks containing 27 imaging voxels (0.73 ml) were defined at the centers of relevant activation/deactivation/correlation clusters to extract the average % BOLD signal from individual contrast maps. These masks were created and centered at the precise coordinates listed in Tables 1, 2, 3, 4, 5. The average and standard deviation values of the BOLD signals within these regions were computed using an in-house program written in IDL. The coordinates of the ROI masks were kept fix across subjects and conditions. Note that the Tables list average statistical values only for clusters that were statistically significant (P<0.05, corrected for multiple comparisons with the random field theory in SPM). Brain regions were labeled using the Talairach daemon (http://www.talairach.org/) [41] and a query range of 5 mm.

**Table 1:** Average statistical significance in regions-of-interest exhibiting BOLD-fMRI activation/deactivation.

						Statistical significance (t-score)			
Brain Region	BA	*X*[mm]	*Y*[mm]		*Z*[mm]	VA	Load	DAT Cau	DAT Put
**VA activation**									
Middle Frontal Gyrus	6	−24		−9	57	**6.5**			
Middle Frontal Gyrus	6	−51		6	39	**4.4**			
Precentral Gyrus	6	−42		−3	48	**4.8**			
Sub-Gyral	6	21		0	51	**13.8**		2.2	
Superior Frontal Gyrus	6	0		12	48	**10.2**			
Superior Parietal Lobule	7	18		−66	57	**10.7**		1.9	
Superior Parietal Lobule	7	−12		−66	57	**9.0**		2.6	
Middle Frontal Gyrus	9	−39		33	30	**4.6**			
Precentral Gyrus	9	36		9	36	**9.3**	2.3	2.0	
Superior Frontal Gyrus	10	24		42	27	**7.4**	2.3		
Superior Frontal Gyrus	10	−36		48	21	**5.7**	1.9		
Middle Frontal Gyrus	11	27		42	−6	**2.3**		−2.3	−2.5
Insula	13	−36		15	9	**5.8**			
Parahippocampal Gyrus	27	6		−30	−6	**7.8**	3.4		−1.9
Inferior Parietal Lobule	40	45		−36	48	**8.3**	2.1		
Inferior Frontal Gyrus	45	−48		33	3	**1.8**			
Cerebellum (Declive)		15		−72	−12	**6.9**			
Cerebellum (Declive)		−24		−63	−21	**6.2**	2.0		
Cerebellum (Nodule)		9		−57	−27	**6.7**	3.0		
Thalamus		9		−21	15	**8.5**	2.9	1.7	
Thalamus		−3		−21	18	**7.5**	2.4	2.1	
**VA deactivation**									
Middle Frontal Gyrus	8	−24		24	45	**−5.1**	−1.9		
Superior Frontal Gyrus	8	−21		36	45	**−4.9**	−1.8		
Insula	13	−33		−21	15	**−11.2**	−2.0		
Insula	13	−54		−36	15	**−5.7**		1.9	
Cuneus	18	−3		−75	27	**−14.8**	−2.6		
Middle Occipital Gyrus	18	27		−87	0	**−4.0**			
Anterior Cingulate	24	−6		33	12	**−6.6**	−2.6		
Parahippocampal Gyrus	28	21		−18	−15	**−3.6**		−1.7	
Paracentral Lobule	31	6		−9	45	**−8.2**	−2.5	1.9	2.1
Precuneus	31	−3		−63	21	**−14.7**	−2.0		
Precuneus	31	−3		−51	33	**−12.1**	−2.2		1.8
Anterior Cingulate	33	−6		18	21	**−3.9**	−2.2		
Angular Gyrus	39	48		−69	36	**−2.9**			
Transverse Temporal Gyrus	41	−54		−18	15	**−8.5**	−3.0		
Inferior Frontal Gyrus	47	−27		30	−3	**−3.1**			
Claustrum		30		−18	15	**−9.1**	−2.1		
Putamen(Lentiform Nucleus)		33		−12	3	**−8.5**	−2.3		

VA: Visual attention; Load: VA-load; DAT Cau/Put: Correlation between BOLD-fMRI signals in the ROI and DA transporters in caudate/putamen. Isotropic cubic ROIs (volume 27-voxels; 0.73 cc) centered at the Talairach coordinates (X, Y, Z). Empty cells are non significant.

**Table 2:** Average statistical significance in regions-of-interest exhibiting VA-load activation/deactivation.

					Statistical significance (t-score)			
Brain Region	BA	*X* [mm]	*Y* [mm]	*Z* [mm]	VA	Load	DAT Cau	DAT Put
**VA-load activation**								
Superior parietal lobe	7	−18	−66	42	2.9	**2.2**		
Thalamus		6	−18	18	7.9	**2.8**		−1.6
Midbrain		9	−30	−9	6.1	**3.1**		−2.6
Thalamus		−9	−21	15	6.8	**2.6**	3.4	
Superior Frontal Gyrus	10	21	45	27	6.7	**2.4**		
Medial Frontal Gyrus	10	15	57	15	3.2	**2.1**	−2.3	−2.3
**VA-load deactivation**								
Cingulate Gyrus	24	−6	−6	36	−6.2	**−3.8**		
Paracentral Lobule	31	6	−12	45	−8.1	**−2.7**	1.6	
Paracentral Lobule	5	0	−27	51	−4.5	**−2.2**		
Anterior Cingulate	32	−6	45	0	−4.0	**−2.2**		
Anterior Cingulate	24	−3	33	9	−6.2	**−2.6**		
Superior Temporal Gyrus	22	48	−15	0	−7.8	**−3.0**		
Precentral Gyrus	13	48	−12	12	−8.3	**−3.6**		
Putamen(Lentiform Nucleus)		33	−12	0	−7.8	**−2.4**		1.7
Postcentral Gyrus	3	−57	−15	24	−3.8	**−2.0**		
Inferior Parietal Lobule	40	−60	−36	24	−2.8	**−1.9**		
Cuneus	19	12	−75	33	−8.1	**−2.7**	−2.7	
Posterior Cingulate	31	−12	−63	15	−11.7	**−2.1**		
Cuneus/Precuneus	31	6	−57	27	−11.0	**−2.1**		
Superior Occipital Gyrus	19	−33	−81	33	−7.1	**−2.1**		
Precentral Gyrus	4	48	−12	36	−4.8	**−2.1**		
Posterior Cingulate	30	18	−54	12	−7.4	**−2.7**		
Paracentral Lobule	31	6	−12	45	−8.1	**−2.7**	1.6	

VA: Visual attention; Load: VA-load; DAT Cau/Put: Correlation between BOLD-fMRI signals in the ROI and DA transporters in caudate/putamen. Isotropic cubic ROIs (volume 27-voxels; 0.73 cc) centered at the Talairach coordinates (X, Y, Z). Empty cells are non significant.

**Table 3:** Average statistical significance in regions-of-interest exhibiting correlations between BOLD-fMRI signals and DA transporters in caudate.

						Statistical significance (t-score)			
Brain Region	BA	*X* [mm]	*Y* [mm]		*Z* [mm]	VA	Load	DAT Cau	DAT Put
**Positive VA-DAT(caudate) correlation**									
Postcentral Gyrus	3	33		–33	48	3.0		**4.4**	2.5
Postcentral Gyrus	3	33		–33	48	3.0		**4.4**	2.5
Postcentral Gyrus	5	30		–39	57	3.2		**4.4**	
Postcentral Gyrus	5	30		–39	57	3.2		**4.4**	
Superior parietal lobe	7	18		–51	57	5.3		**4.5**	2.5
Superior parietal lobe	7	–9		–63	51	4.3		**4.4**	2.6
Precuneus	7	–15		–60	36	–5.4		**2.8**	2.8
Cuneus	17	15		–87	9	1.7		**3.5**	2.1
Middle Occipital Gyrus	18	–30		–87	6	–1.8		**2.5**	2
Cuneus	19	15		–87	21	–2.1		**2.9**	1.9
Posterior Cingulate	30	–27		–72	6			**3.5**	2.6
Sub-Gyral	40	–24		–39	51			**3.7**	2.9
Transverse Temporal Gyrus	41	–33		–27	9	–7.4		**2.8**	2.8
Thalamus		–9		–18	15	6.4	2.6	**3.8**	
Thalamus		–6		–24	3	4.3	2.1	**3.4**	
**Negative VA-DAT(caudate) correlation**									
Superior parietal lobe	7	–24		–69	48	2.4		**–3.1**	–2.9
Middle Frontal Gyrus	11	27		42	–6	2.3		**−2.3**	–2.5
Middle Frontal Gyrus	11	21		24	–9			**−3.6**	–3.5
Extra-Nuclear	13	–39		6	–9			**−3.0**	–2.2
Superior Temporal Gyrus	13	45		–45	15			**–1.9**	
Fusiform Gyrus	19	27		–57	–9	1.8		**−2.7**	–2.3
Anterior Cingulate	24/32	18		39	–6	1.9		**−3.7**	–3.5
Angular Gyrus	39	–33		–57	36			**−2.4**	–2.4
Middle Temporal Gyrus	39	45		–75	21			**−2.6**	–2.3
Inferior Parietal Lobule	40	–39		–51	42	2.7		**−2.3**	–2.9
Claustrum		–36		–6	–6			**−2.4**	–2.4
Hippocampus		–27		–12	–18			**−1.9**	

VA: Visual attention; Load: VA-load; DAT Cau/Put: Correlation between BOLD-fMRI signals in the ROI and DA transporters in caudate/putamen. Isotropic cubic ROIs (volume 27-voxels; 0.73 cc) centered at the Talairach coordinates (X, Y, Z). Empty cells are non significant.

**Table 4:** Average statistical significance in regions-of-interest exhibiting correlations between BOLD-fMRI signals and DA transporters in putamen.

						Statistical significance (t-score)			
Brain Region	BA	*X* [mm]	*Y* [mm]		*Z* [mm]	VA	Load	DAT Cau	DAT Put
**Positive VA-DAT(putamen) correlation**									
Postcentral Gyrus	3	24		–33	45			2.8	**3.0**
Postcentral Gyrus	4	12		–33	57	–6.7	–2.5	3.0	**2.7**
Cuneus	18	–6		–96	21				**1.9**
Posterior Cingulate	30	42		–24	45			2.8	**2.5**
Angular Gyrus	39	–30		–72	9			3.3	**2.8**
Middle Temporal Gyrus	39	–39		–75	30	–3.9		2.1	**2.7**
Supramarginal Gyrus	40	–54		–51	18	–2.9		2.5	**2.8**
Caudate		–21		–30	21			2.6	**3.8**
Claustrum		–24		–21	18	–3.0		1.8	**3.0**
Claustrum		–33		–66	21	–2.1		2.0	**2.3**
**Negative VA-DAT(putamen) correlation**									
Inferior Parietal Lobe	40	–39		–48	39	2.7		–2.0	**−2.7**
Superior Parietal Lobule	7	–3		–30	75				**−2.6**
Middle Frontal Gyrus	10	30		15	9	6.8		–2.2	**−5.1**
Middle Frontal Gyrus	11	18		36	–6	2.3		–4.4	**−4.4**
Fusiform Gyrus	19	30		–78	–18	2.3			**–2.3**
Posterior Cingulate	29	–3		–51	9	–3.7			**−2.1**
Parahippocampal Gyrus	30	27		42	–6	2.3		–2.3	**−2.5**
Parahippocampal Gyrus	30	12		–39	–3			–2.5	**−3.7**
Parahippocampal Gyrus	30	–21		–75	54	2.1		–1.6	**−1.9**
Parahippocampal Gyrus	30	–9		–45	3	–4.1		–1.9	**−3.3**
Posterior Cingulate	30	–24		–57	12				**–2.2**
Fusiform Gyrus	37	30		–51	–12	1.7		–2.3	**−3.0**
Superior Temporal Gyrus	38	–36		3	–12			–3.3	**−2.5**
Middle Temporal Gyrus	39	48		–75	21			–1.9	**−1.8**
Inferior Parietal Lobule	40	–24		–63	51	6.7	1.7	–2.2	**−2.9**
Inferior Frontal Gyrus	47	–48		12	0	2.6			**–2.3**
Claustrum		–33		–12	–3			–2.3	**−2.8**
Midbrain		12		–30	–9	3.8	2.1	–1.6	**–3.2**

VA: Visual attention; Load: VA-load; DAT Cau/Put: Correlation between BOLD-fMRI signals in the ROI and DA transporters in caudate/putamen. Isotropic cubic ROIs (volume 27-voxels; 0.73 cc) centered at the Talairach coordinates (X, Y, Z). Empty cells are non significant.

**Table 5:** Average statistical significance in regions-of-interest exhibiting correlations between VA-load signals and DA transporters in caudate/putamen.

						Statistical significance (t-score)			
Brain Region	BA	*X* [mm]	*Y* [mm]		*Z* [mm]	VA	Load	DAT Cau	DAT Put
**Positive VA Load-DAT(caudate/putamen) correlation**									
Precuneus	7	9		–57	33	–7.6		**2.7**	
Medial Frontal Gyrus	9	–3		51	15			**2.3**	
Middle Frontal Gyrus	10	–24		54	18		1.8	**4.0**	
Cuneus	17	18		–84	9			**2.6**	1.6
Lingual Gyrus	18	–9		–66	3	–4.4		1.9	**2.7**
Cuneus	30	12		–69	9	–2.5		**2.8**	2.5
Cuneus	30	15		–66	12	–5.5		2.3	**3.2**
Posterior Cingulate	31	–15		–66	15	–10.2	–2.0		**2.2**
Anterior Cingulate	32	–3		45	3	–3.8	–2.1	**1.8**	1.9

VA: Visual attention; Load: VA-load; DAT Cau/Put: Correlation between VA-load signals in the ROI and DA transporters in caudate/putamen. Isotropic cubic ROIs (volume 27-voxels; 0.73 cc) centered at the Talairach coordinates (X, Y, Z). Empty cells are non significant.

## Results

**Figure 3:**
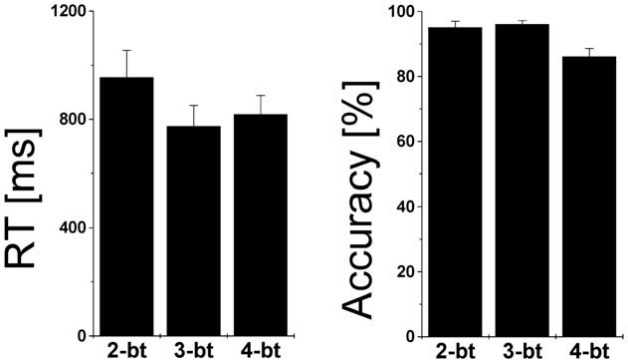
Performance accuracy and reaction time during the parametric VA fMRI task. Accuracy was significantly lower during 4-ball tracking task than during 2- and 3-ball tracking tasks, reflecting the increased difficulty of the task. Values represent means±standard deviations across 14 healthy men.

The performance dataset corresponding to the 4-ball tracking task was lost for one subject due to data acquisition problems. Performance accuracy was higher than 86% for all ball-tracking conditions; it was similar for 2- and 3-balls but was significantly lower for 4-balls compared to 2- or 3-balls (*P*<0.011, paired t-tests), reflecting the increased attention load of the tasks (Fig. 3). However, the effect of increased cognitive load on RT was not statistically significant.

### fMRI: Brain activation

**Figure 4:**
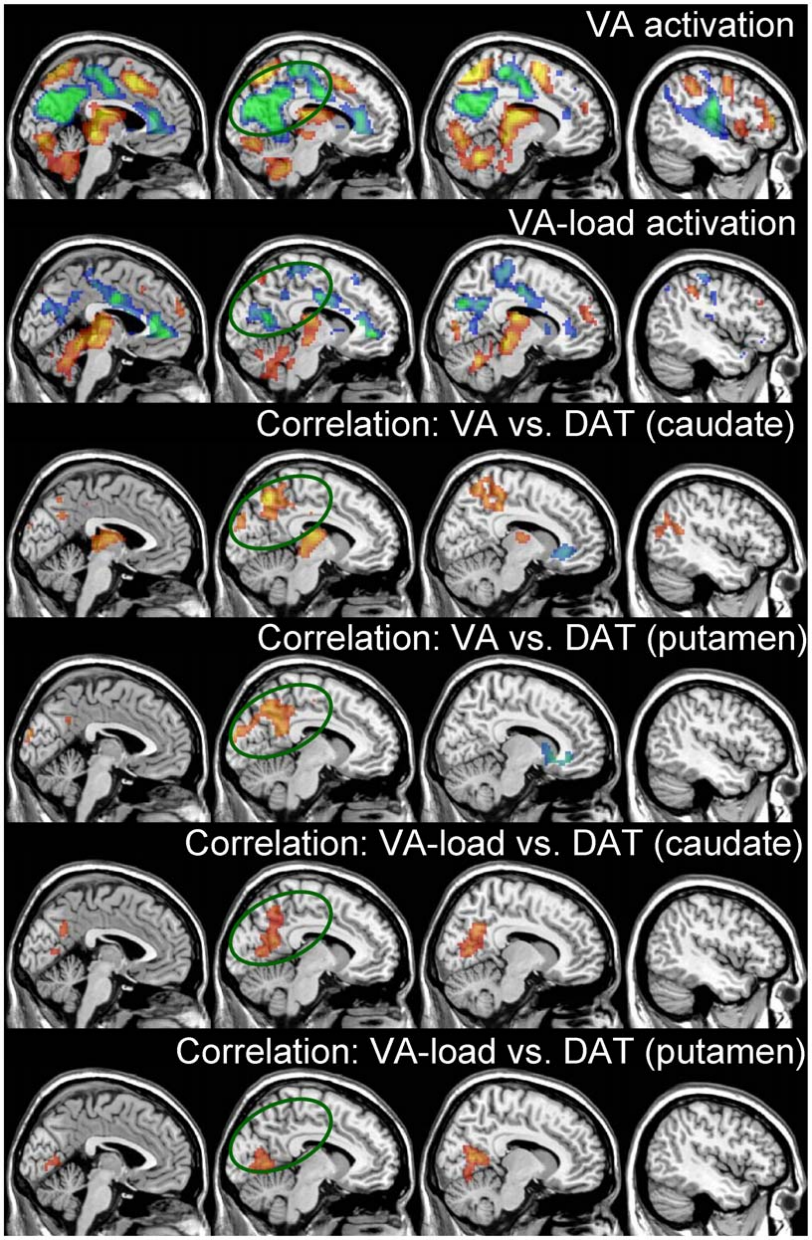
Brain activation and DAT. Sagital views of the brain activation/correlation patterns during VA tasks pattern rendered to a structural MRI image (ANOVA; T-score window = 2.7 to 10; red-yellow: activation; blue-green deactivation). [First row] Average activation and deactivation across all three difficulty levels (2-, 3-, and 4-ball tracking); [second row] increased activation/deactivation caused by increased attentional load (4-ball tracking vs. 2-ball tracking); Statistical maps of positive (red-yellow) and negative (blue-green) correlations between BOLD-fMRI responses in the brain and [^11^C]cocaine (DAT) radiotracer binding in caudate [third row] and putamen [fourth row] as well as VA-load responses and DAT in caudate [fifth row] and putamen [sixth row], rendered to a structural MRI image. Multiple regression (random-effects) analyses. Color maps are t-score windows: 2.7 to 5. The ellipse highlights the DMN regions.

Figure 4 shows that the VA task activated the dorsal network [prefrontal (PFC), superior and inferior parietal, and occipital cortices], thalamus and cerebellum, and deactivated a DMN that comprises ventral precuneus, paracentral lobule, and cingulate cortices [13], as well as insula, prefrontal occipital and temporal cortices, claustrum, putamen, and the parahippocampal gyrus as previously reported [42], [43] (Fig. 4, Table 1). Increased VA load (VA-load), produced higher activation in the thalamus and midbrain and higher deactivation in the DMN, the cingulate cortex (BAs 24 and 32), and left insula (Fig. 4; Table 2). Thus the main activation patterns (VA and VA-load) were consistent with those from previous studies on this VA task [18], [23], [25], [26], [42], [44]–[50].

### DAT-BOLD correlation

**Figure 5:**
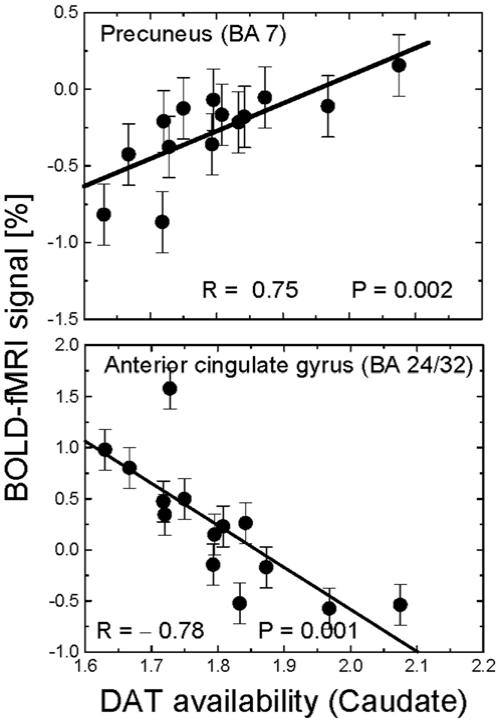
Brain activation in relation to DAT. Linear regression plots of average BOLD-fMRI responses in functional ROIs in the parietal and frontal cortices as a function of [^11^C]cocaine binding (DAT availability) in the putamen or the caudate. R is the Pearson linear correlation factor.

DAT availability in the caudate showed positive correlations with BOLD signals in right postcentral gyrus, bilateral superior parietal lobe (BA 7), left ventral precuneus (BA 7), and left thalamus (*P*_corr_<0.001; Table 3) and negative correlations with those in right perigenual anterior cingulate gyrus (ACG; BA 24/32; *P*_corr_<0.02; Figs. 4 and 5; Table 3); correlations in the thalamus, however, were not statistically significant after removal of one outlier (for this subject, BOLD responses in the thalamus were more than 4 standard deviations above the mean of the other 13 subjects, while the corresponding values of performance accuracy and DAT were within one standard deviation; Figure S1 in supplemental material shows the SPM results with removal of this subject). Similarly, DAT availability in putamen showed positive correlations with BOLD signals in DMN regions (cuneus and ventral precuneus; BAs 18 and 31; *P*_corr_<0.001; Table 4) and negative correlations with BOLD signals in claustrum/putamen (*P*_corr_<0.001; Table 4). Since DMN regions are deactivated during the VA task, a positive correlation between DAT and BOLD signals in these regions reflects less deactivation with higher DAT availability (e.g. a negative correlation with deactivation).

The other clusters in superior temporal and occipital cortices that showed negative correlations between BOLD signals and DAT in caudate and putamen did not survive corrections for multiple comparisons. Most cortical clusters that exhibited positive DAT-BOLD correlations were located at the edges of deactivated brain regions and did not activate/deactivate significantly with VA load (Tables 3 and 4). The supplementary Fig S2 maps these areas with respect to the areas deactivated by the VA task.

In addition, there was a positive correlation between DAT in caudate and VA-load responses in cuneus/ventral precuneus (region increasingly deactivated with increasing VA-load). Overall, the lower the DAT availability in caudate the greater the VA-load deactivation in cuneus/precuneus. The positive correlation between DAT in putamen and VA-load responses in cuneus (area deactivated with increasing load) did not reach statistical significance (*P*_corr_ = 0.08).

## Discussion

Here we show that the availability of DAT in the striatum correlates positively with BOLD signals in precuneus (BA 7) but negatively with those in the ACG (BA 24/32) during VA. We also show a positive correlation between DAT in caudate and VA-load signals in cuneus/ventral precuneus, which were regions that were increasingly deactivated by increased VA-load. Because most of the correlations with DAT occurred in areas deactivated by the task, a positive correlation implies that greater DAT levels were associated with less deactivation (negative correlation with BOLD deactivation).

Dopamine is a neurotransmitter that acts primarily as a neuromodulator changing the efficacy of other transmitter signals as a function of ongoing neuronal activity [51]. For example, DA injected into the striatum led to decreased spontaneous neuronal firing to a greater extent than that of glutamate-stimulated neurons [51]. This effect in neuronal firing is believed to improve signal-to-noise for the detection of task specific neuronal activation in electrophysiological studies [52]. Therefore we predicted that, by decreasing non-task related activity, DA stimulation would result in lower activation of task specific regions (increasing efficiency) and higher deactivation in regions of the DMN, which is deactivated by the task. Specifically, we hypothesized that BOLD signals would be positively correlated with DAT (the lower the DAT the lower the activation in task specific regions and the higher the deactivation in the DMN). The current study corroborates our hypothesis about a positive correlation between DAT and BOLD signals in ventral precuneus/cuneus, regions that belong to the DMN [13]. In addition, the positive correlation between VA-load responses in the DMN and DAT in caudate also confirms our hypothesis that subjects with low DAT (more dopamine) had increased VA-load deactivation in DMN regions. In contrast we identified a negative correlation between DAT and BOLD signals in ventral ACG (BAs 24/32), which is identified by some as being part of the DMN; though it was not a region deactivated by the VA task. These findings are also consistent with our recent study showing that the stimulant methylphenidate, a DAT blocker, reduces the brain metabolic increases induced by a cognitive task (numerical calculations) [17].

### DAT modulation of BOLD responses in ventral precuneus

Subjects with higher DAT availability in striatum had lower deactivation in ventral precuneus (Figs. 4 and 5) and showed a trend to lower deactivation increases with increasing VA-load (Fig. 4) than those with lower DAT availability. Since the ventral precuneus is an area that deactivates during the VA task and is part of the DMN, increased deactivation might reflect that subjects with low DAT deactivated this region and that this effect was greater with increasing attentional load. The precuneus (BA 7), is a major association area that has reciprocal connections with superior and inferior parietal, prefrontal, and occipital cortices as well as subcortical regions (reviewed in [53]). The ventral (precuneus; BA 7) and the dorsal (superior and inferior; BAs 7 and 40) parietal cortices are thought to play an important role in attention shift, and spatial information processing. The precuneus, is involved in alertness [54] and activates in fMRI studies of spatial [23], [42], [55] and orienting [31], [56] attention. Our findings documenting an association with DAT suggest that DA modulates the responses of the precuneus during VA. Though there is some evidence of a scarce DA innervation of the parietal cortices [57], [58] it is more likely that the association between DAT and BOLD responses in precuneus reflects an indirect modulation by DA through thalamo-cortical pathways rather than a direct modulation. Regardless of whether this reflects a direct or an indirect DA modulation, the enhanced deactivation of the ventral precuneus (Fig. 5) in subjects with lower DAT could reflect higher extracellular DA facilitation of attention processing. Conversely, the higher precuneus activation in subjects with higher DAT (i.e., lower extracellular DA) may reflect improper concentration or a compensatory response for improper dopaminergic activity during the VA task. Thus these findings also suggest that subjects who had poorer visual attention performance needed greater usage of the reserve network resources in the precuneus.

### DAT modulation of deactivation in ACG

Brain activation signals in a small region of the ventral ACG (BA 24/32) correlated negatively with striatal DAT availability. Specifically, subjects with lower DAT availability had positive BOLD responses in right ACG whereas those with higher DAT availability had negative BOLD responses (Fig. 5). Note that this area of the ACG was not activated/deactivated by the VA task. The ventral ACG region is one that has been implicated in emotions [11], [59], [60], and one that activates during emotional tasks but deactivates during cognitive tasks [61]. The negative association between BOLD responses in ventral ACG and striatal DAT, suggests that individuals with higher DAT (weaker DA signals) require accentuated deactivation of the ventral ACG, possibly to minimize interference of irrelevant processing (i.e. anxiety or negative emotions like fear in the MRI scanner), in order to sustain attention [59]. The ACG received direct DA innervation from midbrain as well as direct modulation via striato-thalamo cortical pathways [62]. Deactivation in ACG is believed to reflect neuronal inhibition [42], [63] mediated by γ-aminobutyric acid (GABA) interneurons [64]. Indeed, MR studies in humans have shown that the concentration of the GABA neurotransmitter in the ACG predicts negative BOLD responses in fMRI [65]. Since GABAergic synapses have DA D1- and D2-like receptors [66] and dopamine modulates the tonic activity of GABA neurons [67], the negative correlation between striatal DAT and BOLD responses in ventral ACG in the present study suggests a dopamine-mediated enhancement of GABAergic inhibition in the ventral ACG during resting epochs. However, we couldn't assess directly this hypothesis because BOLD-fMRI signals in this study reflect differential responses only (e.g. “tracking” minus “resting”).

In a prior study we showed a positive correlation between DAT and inattention both in ADHD subjects and in controls [15]. We interpreted this finding to reflect the fact that high DAT would lead to lower and shorter lasting DA increases resulting in weaker DA signaling. Indeed using PET we showed that lower DAT availability in humans is associated with higher extracellular DA levels [68]. Because DA signals the saliency value of a stimulus modulating the interest it elicits [15], we postulated that the longer lasting presence of DA in subjects with low DAT would ensure longer lasting interest and attention to the stimuli. Here, we extend the findings to show an association between DAT levels and BOLD activation signals in ACG and precuneus, which are regions involved attention processing. Thus these findings suggest that the association between high DAT availability and inattention reflects the differential regulation of attention networks by DA as a function of DAT levels.

The overall results from this study are also consistent with findings in detoxified cocaine subjects who had decreased DA responsiveness [69], and hyper-activated BA 7 and hyper-deactivated the perigenual ACG (BA 24/32) during the same VA task [26].

These findings have clinical implications both for understanding of the neuropathology in ADHD and the mechanisms of action of stimulant medications that block DAT (i.e., amphetamine and methylphenidate). Prior imaging studies have documented disruption in DAT (reviewed in [15]) as well as in the function and morphology of the ACG and the precuneus in subjects with ADHD [70]–[72]. Our findings would therefore suggest that the beneficial effects of stimulant medications in ADHD might reflect in part their ability to restore the functions in cingulate and precuneus via their dopaminergic enhancing effects.

### Study Limitations

1) This study was based on the assumption that high DAT levels in striatum would result in lower extracellular DA levels and vice-versa. However, we can not rule out the possibility that high DAT levels reflect a denser DA innervation, whereas low DAT could also reflect a decrease in striatal DA terminals, which has been shown in Parkinson's disease [73]. Thus future studies with markers that can differentiate between DA terminal innervation and DAT expression per terminal are necessary to confirm our findings. 2) The relatively small sample size (N = 14) limited the statistical power of the results allowing us to detect regions exhibiting high correlation between transporters and functional signals only. 3) The spatial uncertainty of the fMRI results is not uniform in the imaging volume due to macrovascular [74] and susceptibility effects [75], which vary across MR instruments and pulse sequences, and is influenced by image post-processing steps. Significant image distortion, arising from air/tissue differences in magnetic susceptibility affected the vicinities of the ventral ACG (BA 24/32), increasing the error of the spatial localization up to 17 mm for this cluster. For other brain regions that were activated/deactivated by the VA task (precuneus, superior parietal, occipital and prefrontal cortices, thalamus, and cerebellum), image distortion was significantly smaller and the root-sum-square of all contributions (susceptibility, realignment, normalization, and smoothing) to the spatial uncertainty was lower than 5 mm.

### Summary

We used PET with [^11^C]cocaine to measure DAT availability in the striatum, and BOLD-fMRI to evaluate brain activation during a parametric VA task. Increased DAT availability in striatum, which would result in lower DA in the synapse, was associated with lower deactivation of the precuneus (BA 7) and higher deactivation of the ventral ACG (BA 24/32). Also, increased DAT in caudate was associated with less deactivation in precuneus with increasing attentional load. Thus, this study suggests that lower DAT availability (higher DA signaling), such as that observed in ADHD patients treated with methylphenidate, may facilitate attention by modulating brain deactivation in DMN regions (precuneus).

## Supporting Information

Figure S1 Sagital views of the DAT-BOLD correlation patterns during VA tasks with (top panel) and without (bottom panel) the outlier rendered to a structural MRI image (ANOVA; T-score window = 2.7 to 10; red-yellow). The light-blue circle highlights the thalamic cluster.
(3.44 MB TIF)

Figure S2 BOLD-fMRI activation patterns during VA tasks pattern rendered to a structural MRI image (ANOVA; T-score window = 2.7 to 10; red-yellow: activation; blue-green deactivation). [First row] Average activation and deactivation across all three difficulty levels (2-, 3-, and 4-ball tracking); [second row] increased activation/deactivation caused by increased attentional load (4-ball tracking vs. 2-ball tracking). B: Statistical maps of positive (top row) and negative (bottom row) correlations between BOLD-fMRI responses in the brain and [11C]cocaine (DAT) radiotracer binding in the striatum (caudate and putamen), rendered to a structural MRI image. Multiple regression (random-effects) analyses. Color maps are t-score windows: 2.7 to 5.
(1.40 MB TIF)
