# Supplementary figures and images for: Dopamine Transporters in Striatum Correlate with Deactivation in the Default Mode Network during Visuospatial Attention
Source: PLoS One. 2009 Jun 30;4(6):e6102. doi: 10.1371/journal.pone.0006102 (PMC2699543; doi:10.1371/journal.pone.0006102)

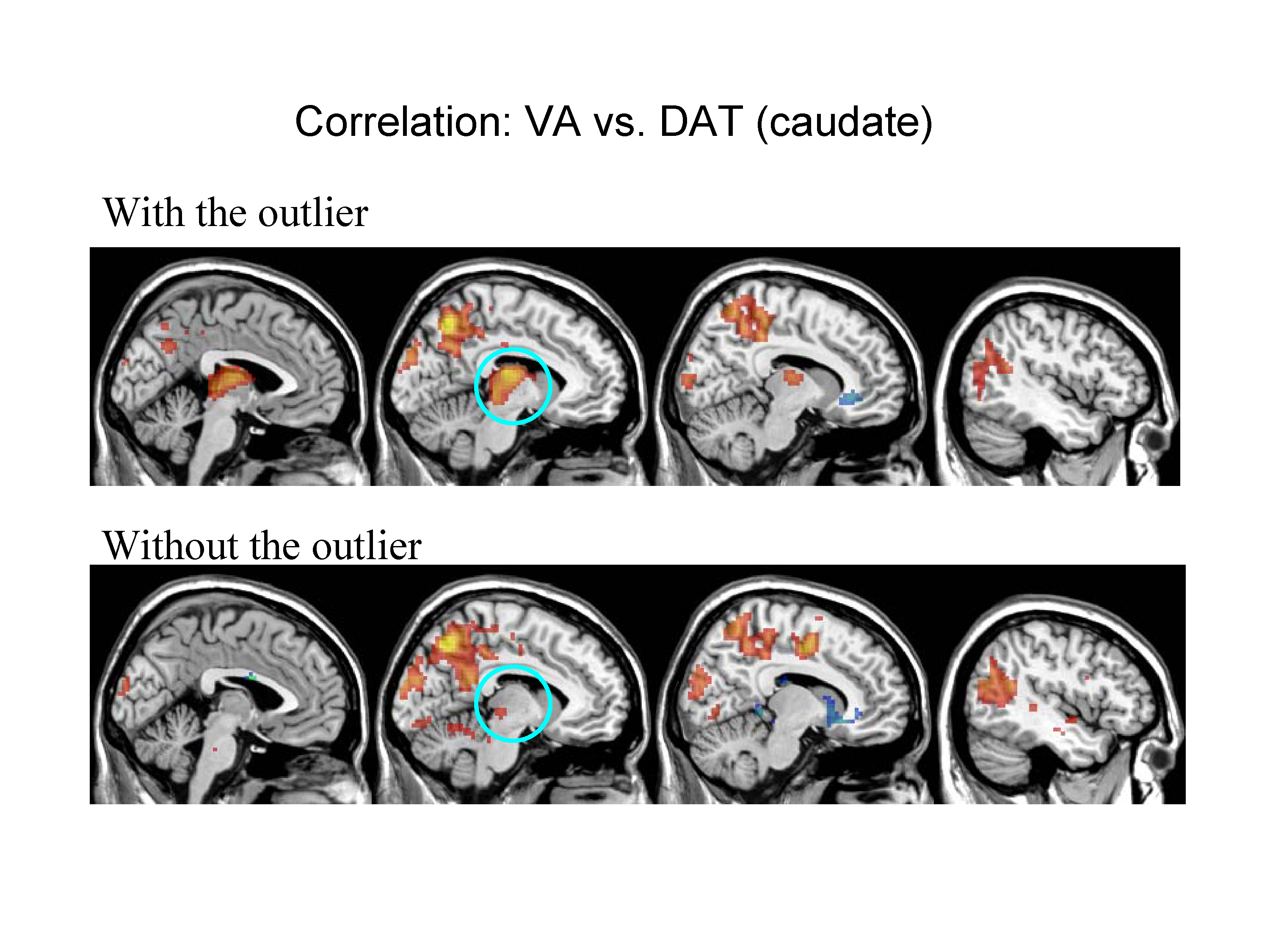

Supplement: Figure S1 — Sagital views of the DAT-BOLD correlation patterns during VA tasks with (top panel) and without (bottom panel) the outlier rendered to a structural MRI image (ANOVA; T-score window = 2.7 to 10; red-yellow). The light-blue circle highlights the thalamic cluster. (3.44 MB TIF) [file pone.0006102.s001.tif]

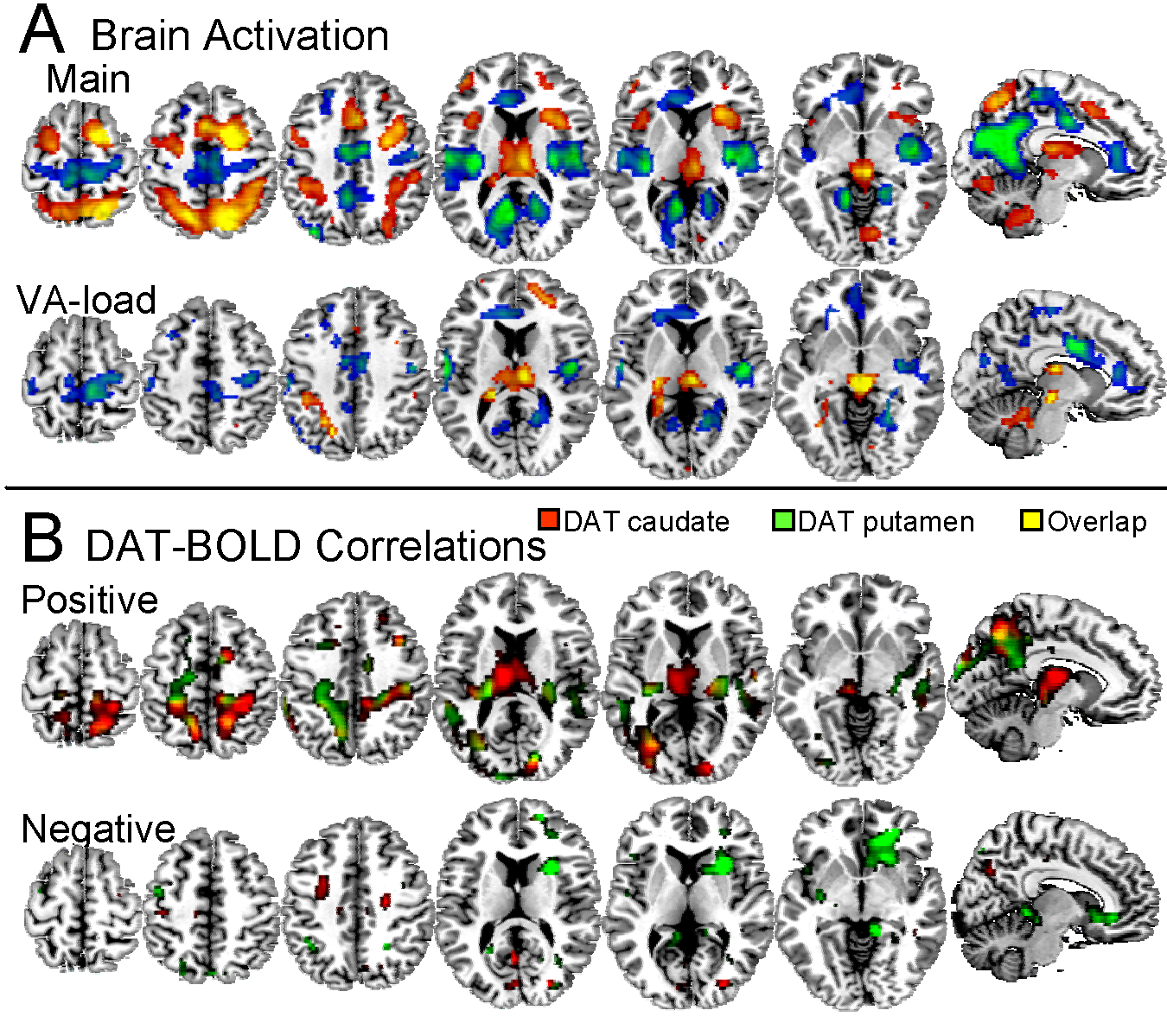

Supplement: Figure S2 — BOLD-fMRI activation patterns during VA tasks pattern rendered to a structural MRI image (ANOVA; T-score window = 2.7 to 10; red-yellow: activation; blue-green deactivation). [First row] Average activation and deactivation across all three difficulty levels (2-, 3-, and 4-ball tracking); [second row] increased activation/deactivation caused by increased attentional load (4-ball tracking vs. 2-ball tracking). B: Statistical maps of positive (top row) and negative (bottom row) correlations between BOLD-fMRI responses in the brain and [11C]cocaine (DAT) radiotracer binding in the striatum (caudate and putamen), rendered to a structural MRI image. Multiple regression (random-effects) analyses. Color maps are t-score windows: 2.7 to 5. (1.40 MB TIF) [file pone.0006102.s002.tif]
